# Spatial–Temporal Dynamics of Adventitious Roots of *Typha domingensis* Pers. Seedlings Grown with Auxin/Cytokinin

**DOI:** 10.3390/life15010121

**Published:** 2025-01-17

**Authors:** Guadalupe Hernández-Piedra, Violeta Ruiz-Carrera, Alberto J. Sánchez, Erika Escalante-Espinosa, Graciano Calva-Calva

**Affiliations:** 1 Programa de Doctorado en Ecología y Manejo de Sistemas Tropicales, Universidad Juárez Autónoma de Tabasco, Carretera Villahermosa-Cárdenas Km. 0.5 S/N Entronque a Bosques de Saloya, Villahermosa C.P. 86150, Tabasco, Mexico; guadalupe.hernandezp@Ujat.mx; 2 Diagnóstico y Manejo de Humedales Tropicales, Universidad Juárez Autónoma de Tabasco, Carretera Villahermosa-Cárdenas Km. 0.5 S/N Entronque a Bosques de Saloya, Villahermosa C.P. 86150, Tabasco, Mexico; alberthoj.sanchez@gmail.com (A.J.S.); erika.escalante@ujat.mx (E.E.-E.); 3 Biotecnología y Bioingeniería, Centro de Investigación y de Estudios Avanzados del IPN, Avenida Instituto Politécnico Nacional 2508, Colonia San Pedro Zacatenco, Ciudad de México C.P. 07360, Mexico

**Keywords:** Typhaceae, in vitro culture, rooting dynamics, rhizotron

## Abstract

The spatial–temporal dynamics of an in vitro radicular system of *Typha domingensis* for the development of rhizofiltration technologies, with the potential for use as a phytotreatment of eutrophicated water, were studied for the first time in the roots of seedlings and in rhizotron systems. The effect of indole-3-acetic acid (AIA) in combination with kinetin (CIN) or 6-benzylaminopurine (BAP) on seedlings cultivated in the light and dark in three radicular systems and in a rhizotrophic regime for the screening of dynamic rhizogenic lines, by weekly allometric measurements of the length and number of roots, were studied. Inhibition of the elongation and branching velocities of roots by BAP and light was observed but CIN increased elongation and branching. In rhizotrons cultivated in light and dark conditions with different AIA/CIN ratios, isolated root explants remained inactive; however, roots attached to a meristematic base presented a significant increase in growth development, with values comparable to those of roots attached to seedlings cultivated in light without hormones. The results revealed that six adventitious rhizogenic root lines with basal meristems have the potential for use in a wide range of environmental and innovative applications in phytotreatment technologies involving eutrophicated water.

## 1. Introduction

Technologies based on adventitious roots from emerging aquatic plants that focus on improving and diversifying their phytofiltration capacity to extract excess nutrients from bodies of contaminated water [1,2] could offer ways to achieve rapid and sustainable applications through in vitro rhizogenesis strategies. These technologies can be applied both at natural environmental sites, such as hypereutrophic bodies of water, and in wastewater treatment systems, optimizing the efficiency of phytoremediation systems [2,3]. Importantly, the efficiency of rhizofiltration is determined by redox conditions and microbial interactions through complex removal mechanisms that influence the availability and mobility of nutrients and chemical contaminants [4,5]. For example, plants can release oxygen through their roots in anoxic environments, creating oxidant microzones that facilitate biogeochemical processes favorable for the absorption and adsorption of eutrophicating ions [4]. Under these conditions, real-time experimental evaluation of the adventitious root system is possible without environmental, biotic, or abiotic interference [6]. The unlimited growth of isolated roots offers great potential for removing pollutants through the use of whole plants in artificial wetlands, even using technologies with transgenic plants, which has received much attention [3,7,8]. In vitro rhizogenesis promotes root differentiation and increases genetic variability using plant growth regulators such as auxins (AUXs) and cytokinins (CYTs). It is well-known that AUXs regulate cell division and expansion while CYTs regulate cell division [2]; however, it has been reported that AUXs, such as indole-3-acetic acid (AIA), are also the major promoters of primary roots and a requirement for adventitious root initiation and development [2,9,10]. Furthermore, the effects of these compounds on adventitious root development and nutrient input levels are reportedly regulated by their synergistic or antagonistic effects with CYTs [9,10]. For example, during root development, the receptor of AUX signals on the cell surface of roots activates pleiotropic responses of the hormonal machinery with the participation of the MAPK signaling network, which works simultaneously with both types of plant-growth regulators [11]. Thus, the adventitious root system works dynamically to handle hormonal stress situations through energy savings, in which both the geometry and forces underlie the kinematic changes in time–space dimensions [12]. Therefore, alternative phenotyping methods are needed to understand root dynamic variability [13]. Currently, the value of roots in plant breeding programs is highly important, and many methods are characterized by being nondestructive, allowing for traits to be measured over time in the same plant or a selection of lines with the desired characteristics [14]. As a result, a new protocol for the culture of plant root systems, named rhizotron, has emerged as a powerful tool for studying the time–space growth dynamics of in vitro lines of the adventitious root system [15,16,17]. In the phytoremediation of contaminated water, species of the family Typhaceae are favored because they have an adventitious root system with a high growth rate and display greater absorption of multiple elements [18,19,20,21,22,23]. *Typha domingensis* Pers. is an emerging aquatic plant that represents the natural community in tropical flood zones of Latin America [24] and has been reported to have a high capacity for the phytoremediation of eutrophicated waters due to its roots offering good apoplastic transport and large xylem-vessel diameters [24,25]. Also, like the cultivation of isolated roots of *Typha latifolia* L. and *Schoenoplectus americanus* (Pers.), phytoremediation of water with *Typha domingensis* could induce prolonged rhizogenesis with a high root biomass phenotype [1,25]. In this work, for the first time, the spatial–temporal dynamics of the adventitious root system from *Typha domingensis* roots of seedlings and isolated root explants, with and without a cauline base, were investigated by the cultivation of in vitro rhizotrons to induce maximum rhizogenesis. The influence of the AUX/CYT levels under light and dark conditions on rhizogenesis was also investigated. Under the premise that the cauline base of *Typha* is a center for meristematic activity [26] and an accumulation site of AUXs [27], it was generally hypothesized that correct amounts of AUXs and CYTs—such as AIA, kinetin (CIN), or benzyl aminopurine (BAP)—might improve the production of adventitious roots for in vitro seedlings or rhizotron systems of *Typha domingensis*. The final aim of this work was to select dynamic rhizogenic lines, that is lines producing roots, with potential for the phytoremediation of bodies of eutrophic wastewater to respond to demands for water resource management and efficient sanitation services [28,29].

## 2. Materials and Methods

### 2.1. Isolation and Germination of Typha Seeds

Infructescence of *T. domingensis* Pers. plants were collected from a wetland located in the Grijalva River basin (17°59′9.91 N and 17°57′48.98 W) of the Metropolitan Area of Villahermosa, Tabasco. The plant samples were deposited in the Herbarium of the Universidad Juárez Autónoma de Tabasco under registration No. 35227. The achenes were detached from the infructescence and stored in a desiccator until seed isolation and germination were performed, as reported previously [30,31]. Each germination experimental unit (n = 8), with 20 mL of Murashige and Skoog basal medium [32] at one-tenth ionic strength (MS0.1) and gelled with agar–agar (4% *w*/*v*), was sown with 15–20 seeds.

### 2.2. Cultures in the Rhizotron

**Figure 1:**
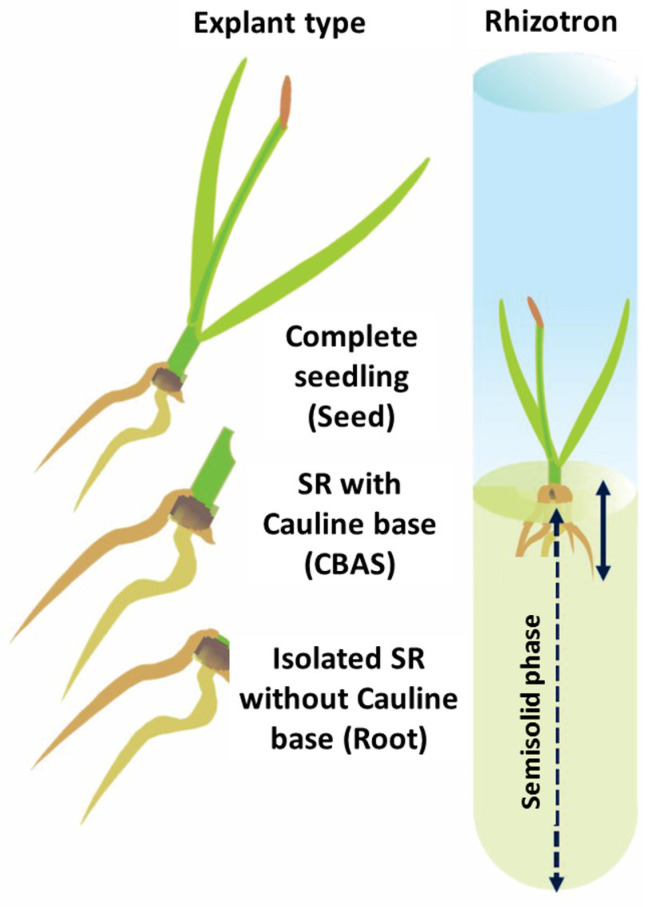
Explant type and rhizotron system used for evaluating the rhizogenesis of *Typha domingensis*. The semisolid phase with the nutrient medium in the rhizotron was covered with 1 mm film of sterile distilled water.

For the establishment of rhizotron cultures, 15-day-old seedlings were removed from the germination unit and used to prepare three sets of cultures according to the explant type (Figure 1): (1) complete seedlings containing up to two leaves and a complete root system (SR), consisting of a seminal root and an adventitious root (SR seedling); (2) an isolated SR with only the cauline base, with 2 mm of the proximal portion of the leaf meristematic base (SR cauline); and (3) an isolated SR without the cauline base (isolated SR). Each explant was transferred to a sterile rhizotron system consisting of 25 × 200 mm test tubes with 25 mL of agar-gelled 0.1 × MS nutrient medium and a 1 mm film of sterile distilled water (Figure 1). The explants were positioned in a photogeotropic position, with the root end introduced one centimeter below the gelled phase of the rhizotron. When needed, AIA (Aldrich I3750 Sigma-Aldrich Merck KGaA, Darmstadt, Germany), BAP (B3408 Sigma-Aldrich Merck KGaA, Darmstadt, Germany), and CIN (K0753 Sigma-Aldrich Merck KGaA, Darmstadt, Germany) from stocks of 1 mg/L were added to the culture medium prior to the sterilization of the rhizotron.

### 2.3. Image-Based Phenotyping of Root Explants Cultivated in Rhizotron

The morphological characteristics of the root system emerging from complete seedlings or their explants cultivated in the rhizotron were quantified by image-based phenotyping of root development, as described by [33] for the roots of *Typha* and by [2,26,34] for adventitious roots of other emerging species. The images of the total root system were captured weekly with a Kodak EasyShare C143 digital camera (Eastman Kodak Company Rochester, NY, USA) through the photographic image tracing method described by [33], and processed with ImageJ V. 1.52h 16 October 2018 [35,36,37] by measuring the total root length (LR) and the total number of roots (NR) to estimate the elongation rate (TE) and branching rate (TRAM), as described for phenotyping by using density-based models [17]. For the allometric measurements, relative growth rate (TCR) was estimated, as reported for the root system of several plant species [38,39,40]; meanwhile, the root length density (DLR) and branching density (DRAM) were estimated, as reported for the characterization of plant species with putative differences in root system size using density-based models [17,40,41]. The formulas used to estimate the phenotyping parameters are detailed below:
**Parameter****Formula****Description****References**Elongation rate (TE) mm d^−1^$TE=\frac{l^{\left(0\right)}}{n^{d}}$*l*^(0)^ Total root length (LR)/days of culture *n*^(d)^
[17]Branching rate (TRAM) Roots d^−1^
$TRAM=\frac{{nr}^{\left(1\right)}}{n^{d}}$*nr*^(0)^ Number of adventitious roots/days of culture *n*^(d)^
[17]Relative growth rate (TCR) mm mm^−1^ d^−1^$TCR=\frac{InLR_{2}-InLR_{1}}{t_{2}-t_{1}}$Growth rate relative to root length at the end of the culture time with regards to the initial root length[38,39,40]Root length density (DLR) mm cm^−3^$DLR=\frac{LR}{v}$Overall length of roots per unit volume root length LR/volume v
[17]Branching density (DRAM) cm^−3^$DRAM=\frac{NR}{v}$Connections between roots (branching points NR) per unit volume branching points/volume v[17]

### 2.4. Effects of Plant Growth Regulators and Light/Dark Conditions

**Figure 2:**
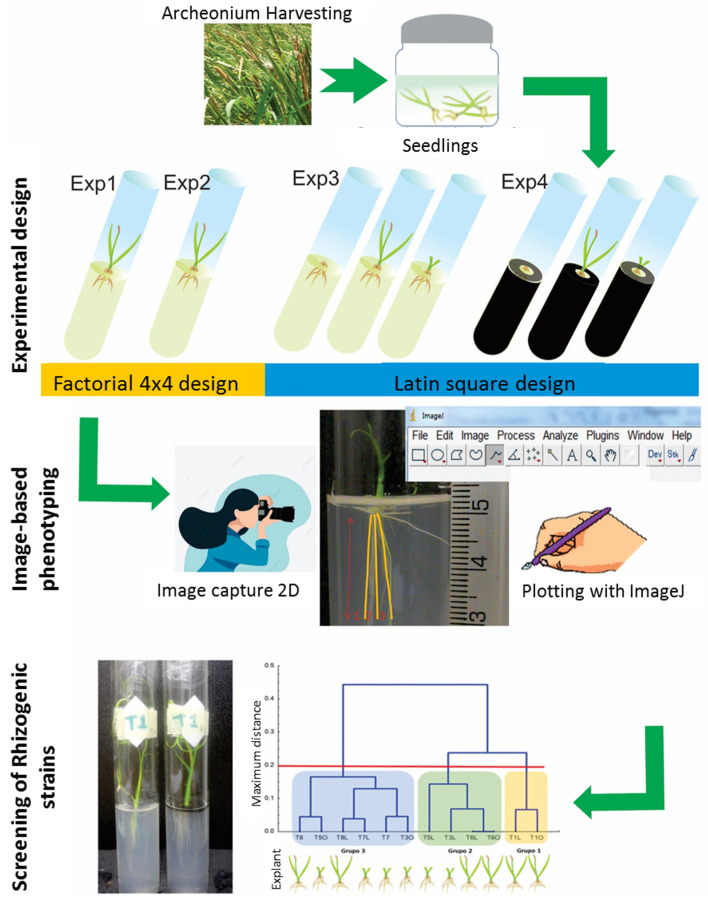
Diagrammatic summary of the overall methodology of the experimental strategy used to evaluate rhizogenesis in cultures of complete *Typha domingensis* seedlings and their explants by phenotyping image-based analysis. Two factorial experimental designs focused on discerning the effects and interaction of AIA and CIN (Exp 1) or AIA and BAP (Exp 2) on rhizogenesis in complete seedlings, and two Latin square experimental designs were used to study the main effects of the concentrations of AIA and CIN and the explant type under light (Exp 3) and darkness (Exp 4) on the rhizogenesis in rhizotron cultures.

The effects of growth regulators and light/dark conditions on the rhizogenesis in the root system of complete seedlings and their explants were examined using the experimental strategy depicted in Figure 2; this was based on changes in the length and number of roots under 28 days of in vitro cultures, as described previously [31], using the image-based protocol described above.

Two 4 × 4 factorial experimental designs—focused on screening the effect of the balance and concentrations of AIA and CIN (Exp 1) or AIA and BAP (Exp 2), each at the levels of 0, 0.1, 1, 10 mg/L, on the rhizogenesis—were applied to complete seedlings cultivated for 28 days under a light/dark photoperiod of 16/8 h with cold light lamps, Phillips, USA, 90 ± 2 μmol photons m^−2^ s^−1^ (Quantum light meter, Spectrum Technologies, Inc. El Paso, TX, USA). Each treatment included six replicates (n = 96) with sampling for phenotyping traits every 7 days.

Further investigation to improve the rhizogenesis in rhizotron cultures was implemented by removing variability due to explant type, the growth regulator concentrations, and illumination conditions. A Latin experimental design was applied to study the main effects of the AIA and CIN concentrations (0, 0.1, 1 mg/L) and the explant type (complete seedling (seed), SR with cauline base (CBAS), or isolated SR without cauline base (root)), resulting in 9 treatments (Table 1).

The Latin square design was executed under light (Exp 3) and darkness (Exp 4) to study the rhizogenesis in rhizotron cultures, as outlined in Figure 2. Each rhizotron was inoculated with an explant from the second batch of in vitro-grown seedlings and five repetitions were performed for each treatment (n = 45). The culture period was 28 days, which allowed for the phenotyping traits (three rates and two densities) to be measured every seven days. Treatments in light (Exp 3) were cultivated under the photoperiod, as indicated above for the factorial designs. For treatments in dark conditions (R-OSC), the rhizotrons were covered with black cardboard (185 g m^−2^), and the water film was covered with a black ethylene–vinyl–acetate ring.

The phenotypic traits used to evaluate the temporal dynamics of root development were TE, TRAM, and TCR, whereas those used to evaluate the spatial dynamics were DLR and DRAM using generally reported protocols [15,16,17,38,39,40,41]. To characterize the optimal time of the phenotypic traits, rhizogenesis was plotted as a function of the growth regulators concentration (Experiments 1 and 2) and the type of explants (Experiments 3 and 4).

### 2.5. Statistical Analysis

For experiments concerning the effect of phytoregulators, tests of normality (Kolmogorov–Smirnov and Cochran C) and homogeneity of variance (Levene’s) were performed for the phenotypic traits (TE, TRAM, TCR, DLR, and DRAM). Data that met the normality assumptions were analyzed with MANOVA and post hoc Fisher’s LSD test. Data that did not meet the above assumptions were analyzed with Kruskal–Wallis ANOVA by ranks. For the Latin square design, ANOVA was applied in Experiments 3 and 4. The statistical program selected the Latin square design at random, a principal factor (explant), and block factors (concentrations of AUX and CIN), each with the same number of levels. The explant was the variable evaluated in the Latin square design. The selection of hyperrhizogenic lines was performed by the method of clusters of the farthest neighbor complete linkage (maximum distance or minimum similarity). TE, TRAM, TCR, DLR, and DRAM data from Experiments 3 (R-LUZ) and 4 (R-OSC) and from the matrix sum R-LUZ + R-OSC were used. The criterion for assigning the number of groups was carried out with the geometric morphometrics amalgamation method [42,43]. The statistical significance was *p* < 0.05, and all analyses were performed using Statistica V8 software (Stat Soft Inc., Tulsa, OK, USA).

## 3. Results

### 3.1. Effect of Phytohormones on Dynamics of Rhizogenesis of Typha domingensis Seedlings

**Figure 3:**
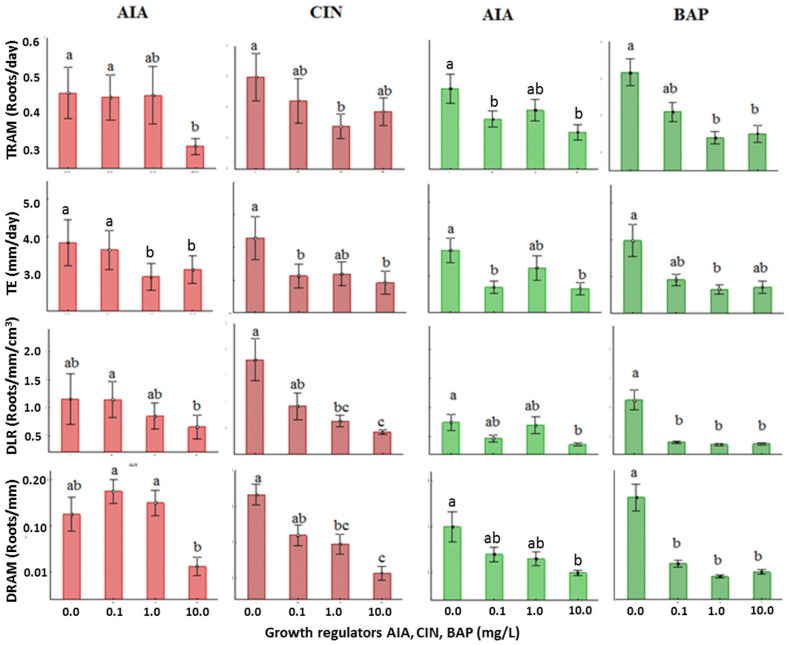
Effect of the concentration of growth regulators on the phenotyping traits of root systems of complete seedlings cultivated under a light/dark photoperiod of 16/8 h by 7 days for the branching rate (TRAM) and the elongation rate (TE), and by 21 days for the branching density (DRAM) and the root length density (DLR). Data represent the corresponding means for each growth regulator level from 4 × 4 factorial experimental designs to study the effect of the balance and concentrations of AIA/CIN (Exp 1) and AIA/BAP (Exp 2). Treatments included six replicates. Error bars represent the SD from each factor level mean (n = 24) from the 16 treatments. Different letters above the error bars indicate significant differences at the 0.05 level (ANOVA and post hoc Tukey’s multiple range test).

Proper amounts and the balance of AUXs/CYTs, such as AIA and CIN or BAP, are known to promote organogenesis in plant tissue and organ cultures [9,10,11]. Thus, with the goal of improving the production of adventitious roots in *Typha domingensis* seedlings, the influence of the concentration and balance of AIA/CIN (Exp 1) or AIA/BAP (Exp 2) was investigated for 21 days of culture in rhizotrons using factorial experimental designs (Figure 2). The phenotyping traits (TE, TRAM, TCR, DLR, and DRAM) of roots in the SR from seedlings of *Typha domingensis* cultivated in rhizotrons under a light/dark photoperiod of 16/8 h were significantly affected (*p* < 0.05) by both the concentration and the balance of the growth regulators (Figure 3), producing significant effects on the total root length, LR, and number of roots, NR (*p* < 0.01 to 0.05), and on the TE, TRAM, TCR, DLR, and DRAM phenotypes (Table 2 and Table 3). Invariably, all phenotypes displayed higher values when the concentrations of both growth regulators were lower. Notably, severe inhibitory effects were observed in the presence of 10 mg/L of any growth regulator.

**Figure 4:**
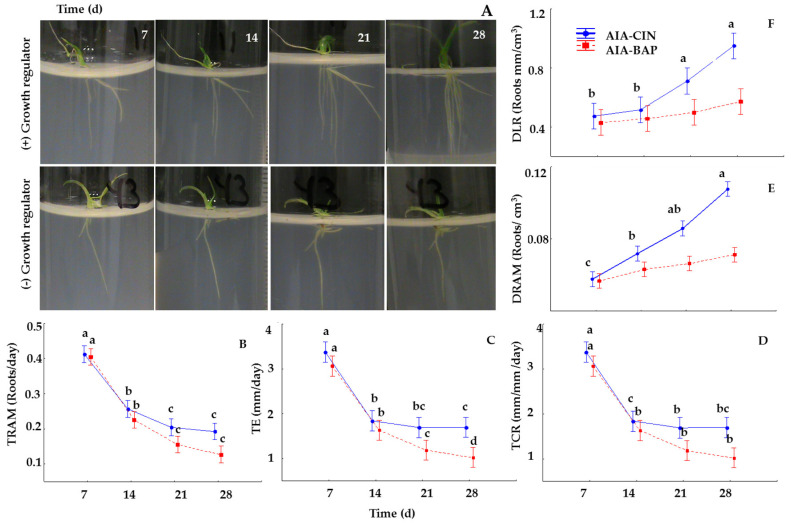
Weekly dynamics of phenotyping the root system development of seedlings of *Typha domingensis* cultivated for 28 days in rhizotrons under photoperiods to study the effect of the balance and concentrations of AIA/CIN (Exp 1) and AIA/BAP (Exp 2) by factorial experimental designs. (**A**) frames of representative pictures contrasting the growth pattern and weekly phenotyping of roots in seedlings cultivated without any growth regulator (−) versus those cultivated with 10 mg/L of CIN (+). Dynamic curves (**B**–**F**) show the weekly changes in the overall mean value from the factorial design conducted with AIA and CIN (blue lines), and that conducted with AIA and BAP (red lines) on the branching rate (**B**), total elongation rate (**C**), growth rate (**D**), branching density (**E**), and root length density (**F**). Error bars represent the SD of the weekly overall means from the 16 treatments with 6 replicas (n = 96). Different letters on the error bars indicate groups of means with significant differences at the 0.05 level (ANOVA and Tukey’s multiple range test).

The weekly average for phenotyping traits—such as TE, TRAM, TCR, DLR, and DRAM—of roots in the SR from seedlings of *Typha domingensis* cultivated in rhizotrons under a light/dark photoperiod of 16/8 h (R-LUZ) were also significantly affected (*p* < 0.05) by both the concentration and the balance of the growth regulators (Figure 4A,F). The TE, TRAM, and TCR showed the maximum response after 7 days of culture (Figure 4B–D), whereas DRAM and DLR were better after 28 days (Figure 4E,F). With respect to the AIA/CIN and AIA/BAP balance, the graphical representation of the average rhizogenesis between rates and densities stood out, particularly with AIA/CIN (Figure 4B–F). The rates (TE, TRAM, and TCR) decreased from the second week (*p* = 0.001), and the decrease was greater in the presence of BAP than in the presence of CIN at week four (*p* = 0.001). The DRAM and DLR of the AIA/CIN increased, doubling the initial averages for the fourth week (*p* = 0.001). However, with AIA/BAP, the densities did not significantly differ at the sampling times (Figure 4E,F).

**Figure 5:**
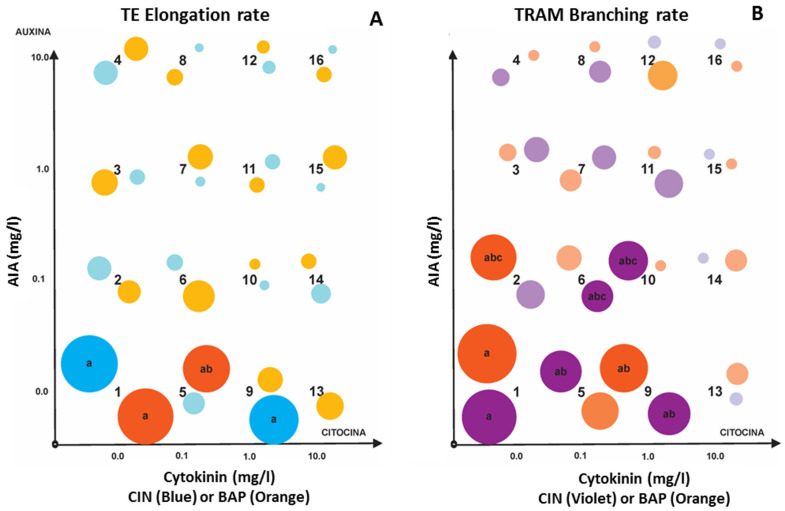

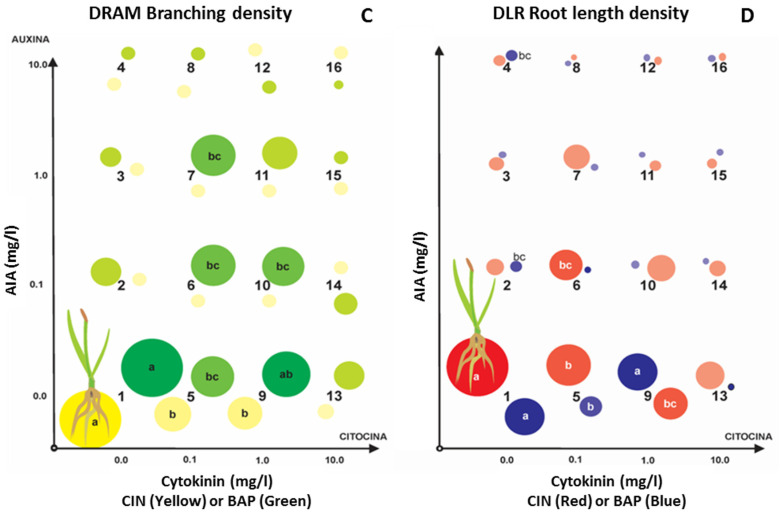
Effect of the balance and concentrations of AIA/CIN (Exp 1) and AIA/BAP (Exp 2) by complete 4 × 4 factorial experimental designs (1–16 dots) on the phenotypes related to the growth rate (TE (**A**) and TRAM (**B**)), and the contact surface (DRAM (**C**) and DLR (**D**)) parameters of the root system of *Typha domingensis* seedlings cultivated for 28 days in rhizotrons under photoperiods. Different letters in the dots (size and color intensity are related to the mean value) indicate means with significant differences at 0.05 level (ANOVA and Tukey’s multiple range test).

With respect to the phenotypic traits DLR and DRAM—associated with the root surface that may come in contact with any external material, named contact surface—and those associated with growth rate (TRAM and TE), rhizogenesis was significantly affected (*p* < 0.05) by both the AUX/CIN (Table 2) and the AUX/BAP (Table 3) combinations. In the absence or low content of AIA and 0–1 mg/L of any CYT, root phenotypes with relatively high growth rates and high densities were produced (Figure 5, Table 2 and Table 3). For growth-rate-associated phenotypes, in the combinations of 0.1 mg/L AUX/0 mg/L CIN and 1 mg/L AUX/0 mg/L BAP, the TE was significantly greater (Figure 5A). Similarly, for TRAM, combinations of 0.1 mg/L AUX/0 mg/L CIN, 0.1 mg/L AUX/0.1 mg/L CIN, 1 mg/L AUX/0 mg/L CIN, 1 mg/L AUX/0.1 mg/L CIN, 1 mg/L AUX/0 mg/L BAP, and 0.1 mg/L AUX/0.1 mg/L BAP produced greater branching (Figure 5B,C). Conversely, in treatments with BAP (Table 3), although the tendency of the branching (TRAM and DRAM) was similar to that of the elongation rate TE (Figure 5A vs. Figure 5B,C), for the TE, the 0.1 mg/L AUX/0.1 mg/L CIN combination was significantly greater (Figure 5C, Table 2).

With respect to the phenotypic traits associated with the contact surface, DRAM was significantly greater in the absence of phytoregulators (Figure 5). The combination of 1 mg/L AIA with 0–1 mg/L of any CYT resulted in the increased growth and yield of roots (Table 2 and Table 3). For DLR, for example, the best average value was found in rhizotrons without phytoregulators and was significantly different by 1.64 times from the second-best value observed at 0.1 mg/L AUX/0 mg/L CIN (Figure 5D). Interestingly, with respect to the BAP treatments, no significant differences were detected, and the combination of 0 mg/L AUX/0 mg/L BAP produced the highest average values for most phenotype traits (Table 3).

Thus, in terms of growth rate and density, a lower inhibitory effect on adventitious root induction in seedlings was observed in the treatments with 0–1.0 mg/L of AIA in combination with 0–1.0 mg/L of CIN or BAP (Table 2 and Table 3). Severe inhibitory effects of both phytoregulators on rhizogenesis were observed in combinations of AIA with CIN or BAP at 10 mg/L each, which was significantly stronger for the presence of any CYT than for AIA. For example, the average values for the relative growth rate (TCR) were in the range of 0.26–0.16 mg/L CIN, whereas for BAP, the range was from 0.25 to 0.02. Notably, with BAP, the TCR decreased up to 12.5-fold, whereas with CIN the decrease was 1.6-fold. A similar trend was observed for the DRAM phenotype, in contrast to the root length density, the average root length density was 7.5 times greater in the presence of CIN and 5.25 times greater in the presence of BAP.

### 3.2. Effects of Growth Regulators, Explant Type, and Light Conditions on the Spatial–Temporal Dynamics of Phenotyping of the Root System

**Figure 6:**
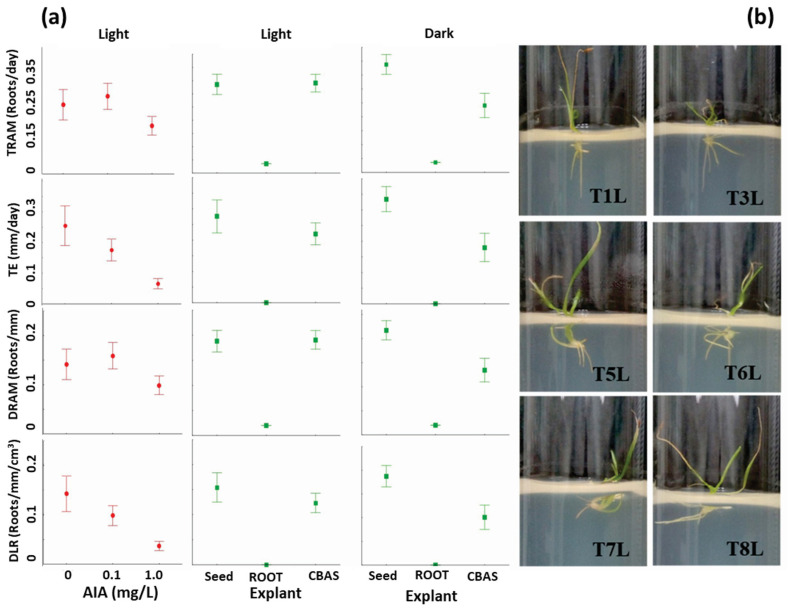
(**a**) Changes in the means of the spatial–temporal dynamics of phenotypes of adventitious roots in explants cultivated in rhizotron under the Latin square experiments in light (Exp 3) and darkness (Exp 4) to evaluate the effect of the AIA and CIN concentrations and the explant type (complete seedling (seed), isolated SR without cauline base (root), and SR with cauline base (CBAS); and (**b**) frames of representative pictures highlighting the weekly phenotyping of the branching density DRAM (**left**) and branching rate TRAM (**right**) of the explants cultivated with seedlings (T1, T6, T8), and CBAS (T3, T5, T7) explants, and 0, 0 (T1), 0, 1 (T3), 0.1, 0.1 (T5), 0.1, 1 (T6), 1, 0 (T7), 1, 0.1 (T8) mg/L of CIN, AIA, and under light (L) conditions. TE = elongation rate; DLR = length density.

Based on the above results, the main effects of the AIA and CIN concentrations and the explant type (complete seedling, SR with cauline base, and isolated SR without cauline base) on the spatial–temporal dynamics of adventitious roots were further investigated by applying two Latin square designs, consisting of testing the medium cultures prepared with each concentration of AIA with each concentration of CIN and inoculated with each explant type, resulting in nine treatments (Table 4). A complete set of treatments was performed under light conditions (Exp 3) and the other complete set under dark (Exp 4) conditions. After 28 days of culture, the phenotyping traits for the evaluation of the root system development were assessed. As shown in Table 4 and Figure 6, growth and phenotyping of roots were severely affected by the explant type, growth regulators, and light regime. As observed in Figure 6, the isolated roots without the cauline base (isolated SR) did not survive under any culture conditions, therefore, it was concluded that they were nonfunctional explants.

In cultures under light conditions (Table 4, Exp 3), the AIA concentration and type of explant affected both the phenotypic traits for evaluation of the temporal dynamics of SR growth or development (TE, TRAM, and TCR), and those for evaluation of the SR density or spatial dynamics (DLAR and DRAM). However, the concentration of CIN did not affect any phenotypic trait, with values similar to those observed in the results of the AIA/CIN factorial experiments (Table 2).

The effect of the explant type and growth regulators in the dark were similar to those in the light (Table 4); however, the presence of light positively affected the dynamic variables in the seedling explants, which were significantly greater than those in the root systems of cauline explants (Table 4). A positive effect of low concentrations of AIA on root growth was evident (Figure 6). While for 0 and 0.1 mg/L AIA, the dynamic parameters were statistically similar, lower average values were observed with 1 mg/L AIA, similar to the effect observed in the above results from the factorial experiment (Figure 5). Notably, in treatments with roots with cauline explants, the stimulation of the rate and density of branching was greater with AIA and CIN (0.01 mg/L) under light conditions; however, complete seedling explants presented the highest values for most phenotypic parameters in light and darkness.

### 3.3. Selection of Rhizogenic Lines Based on Four Traits from the Phenotyping Image Data of the Root System

**Figure 7:**
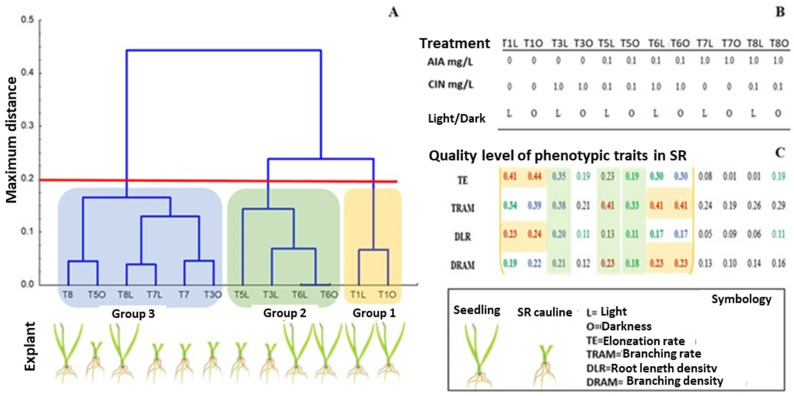
The 12 adventitious rhizogenic root strains of in vitro cultures of *Typha domingensis* seedling explants were grouped based on the quality level of the 4 phenotypic traits examined by image analysis of their root system. (**A**) Dendrogram of the rhizogenic strains; (**B**) identity regarding cultivation treatment; (**C**) matrix of the quality level (averages) of the phenotypic traits: high (red), medium (blue), and low (green).

Based on four phenotyping traits from the image data of the root systems, several adventitious rhizogenic strains showing dynamic growth were selected (Figure 7). Among the most dynamic rhizogenic strains from the cluster groups, six were distinguished by their superior phenotypes in length and branching. Among these, the SR of the caulinar explants competed with those from the complete seedling explants for any of the four estimated phenotypic traits. Under the light condition (L), three groups were found at a level of 0.15. Group 1 included a single rhizogenic line (T1L) of complete seedlings cultivated without growth regulators, resulting in a phenotype with relatively high TE and DLR. In Group 2, the most common phenotypes were TRAM and DRAM, with three rhizogenic strains (T3L, T6L, and T5L) cultivated with the lowest concentration of AIA. Group 3, with two rhizogenic strains (T7L and T8L), was distinguished by being cultivated with relatively high concentrations of AIA. Conversely, under dark conditions at the same quality level of 0.15, four groups were found. The first group included the rhizogenic strain T1O, cultivated without a growth regulator, which presented the greatest elongation average, and in the second group, the T6O rhizogenic strain cultivated with a combined concentration of AIA/CIN (0.1/1 mg/L) presented the greatest branching average. Both strains were obtained from complete seedling explants. The third group included two rhizogenic strains characterized by SR with cauline base explants cultivated in the presence of a single high concentration of AIA (T7O) or CIN (T3O). The fourth group included rhizogenic strains cultivated with combined amounts of growth regulators and was characterized by the lowest average values of the quality variables.

The clustering pattern when the two previous matrices in light and dark conditions were merged (Figure 7C) resulted in groups at a level of 0.2. Group 1 included two rhizogenic lines, T1L and T1O, from SR seedling explants without growth regulators. Group 2 included the rhizogenic lines T6O and T6L from complete seedlings cultivated with low combinations of AIA and high CIN. The SR from explants with the cauline base produced the R3L when cultivated in the light condition with high concentrations of CIN, and the T5L line when cultivated with low concentrations of AIA and CIN. The third group included the T3O line produced by the SR of explants with cauline base cultivated with high concentrations of CIN, and the T7O, T7L, T8L, T5O, and T80 strains when cultivated with high concentrations of AIA. Group 1 included rhizogenic lines expressing a phenotype with higher TEE and DLR. In contrast, Group 2 presented higher TRAM and DRAM values. In total, the most dynamic strains were T1O, T6O, T1L, T3L, T5L, and T6L, which presented the fastest and densest root phenotypes; however, the phenotypes of the T6O and T1O lines presented greater growth rate and density, with the first phenotype corresponding to branching and the second corresponding to elongation of the SR of complete seedlings. The second-best root phenotypes of complete seedlings were found for the T1L and T6L strains. Notably, the rhizogenic lines T3L, T5L, and T5O from the SR from explants with a cauline base presented the second-best DRAM and TRAM values.

## 4. Discussion

In the rhizotron cultures, the growth regulators and light conditions for dynamic adventitious root strains of *Typha domingensis* were defined. As expected, the cultivation of isolated roots was achieved when the explants remained attached to the cauline base and when complete seedlings were used. This observation is consistent with reports that the cauline base can provide the root system with endogenous AUXs, promoting its growth development [44]. However, the inactivity of isolated root explants without the cauline base, even in the presence of growth regulators, was unexpected. This result contrasts with reports for in vitro cultures or roots of *Typha latifolia* [1,25]. The contrasting results might be because, for those reports, mature root segments were cultivated in MS liquid culture medium supplemented with 40% more salts and sucrose, and half of the light intensity than that employed in the present work. Under these culture conditions, they reported thinning of the cell wall of the roots, which decreased the capacity to absorb metals. In this work, it is assumed that isolated root explants were susceptible to the availability of salts because of the superficial biofilm in the rhizotron system. Additionally, with isolated root explants, the aerenchyma is disconnected and the transport of O_2_ from aerial tissue is no longer possible since the network of lagoon spaces is continuous in the direction of leaves to roots and is formed during the normal development of the plant [45]. Other differences occur in rhizogenic responses, even among the same plant species, through tissue-specific AUX homeostasis and recalcitrant signals of rooting [44].

In seedlings as explants, the autoregulatory process of the radical system was altered by the inhibitory effects of exogenous growth regulators. This observation suggests that the addition of phytoregulators was avoidable, which contrasts with reports for several Typhaceae species where an increase in biomass with increasing concentrations of AIA has been reported [1,25]. The weak positive effect of CIN at high concentrations as a rhizogenic stimulant was notable; however, the inhibition observed in the rhizogenesis process with growth regulator was in the order AIA > CIN > BAP. In particular, a greater deceleration of growth rate, density of elongation, branching density, and a greater relative growth rate in treatments with BAP than with CIN were evident. Additionally, the increases in branching and elongation densities in the presence of CIN at low concentrations were specific for seedling explants (Figure 3 and Figure 4). Consequently, the selection of rhizogenic lines under the criterion of using complete seedlings with the lowest concentrations of AIA and CIN is acceptable. With this decision, Latin square designs produced a more precise evaluation of the growth rate and density of the root systems and less variability in the allometric variables of branching and elongation, commonly used in different studies.

According to the experimental objective, the expected differences at lower concentrations of AIA and CIN were evidenced in the presence of light with the explants that had integrated the cauline bases, as the growth of totally isolated root explants was inoperative. In light conditions, the speed and density of branching of the cauline root–base system exceeded those of the seedlings, although the rate and density of elongation were better with complete seedlings; however, in dark conditions, these explants produced better values for the whole phenotypic parameters in complete independence of AUXs. These parameters indicate that the contribution of CIN improved the rates and densities of branching of the explants. These results ae compatible with theories indicating the interference of CYTs in the exogenous regulatory network of radicular systems and are the first to be cited for *Typha* from the perspective of dynamic function. Although it has been widely demonstrated that the growth of roots is activated by AUXs—especially AIA in whole plants, root cuttings, mutants, and transgenic plants that overproduce AIA—many aspects related to the rhizogenic dynamics are unknown for in vitro cultures of adventitious roots cultivated in the presence of AUXs and CYTs, such as AIA, CIN, and BAP; moreover, owing to their antagonistic effects on light and darkness [10], the optimal AUX/CYT ratio should be investigated. For example, it was recently revealed that the length and activity of the root system of *Pteris vittata* increased with an appropriate AIA/CIN ratio [46], as did the proliferation of adventitious roots of *Oryza sativa* with AIA and strigolactone balances [47]. In this work, the combined concentration of AIA/CIN (0/0 or 0.1/1 mg/L) had the best branching average (Table 3). These observations are consistent with the characteristics of continuous rhizogenesis in the absence of phytoregulators for transformed roots of *Lotus corniculatus*, and isolated roots cultivated in continuous growth in which nodules are transformed under light and darkness [6].

For the effective selection of rhizogenic lines, the optimal growth of seedlings and explants of roots with a cauline base under light and dark conditions might be attributed to the direct effect of AIA and marginally to the CIN concentration. The AIA/CIN synergism was effective only with 1.0 CIN and 0.1/0.1 mg/L AIA/CIN, which was notable for both growth rate and density responses as a function of the amount of growth regulator and was less effective for the primary allometric variables of branching and elongation. It was evident that small concentrations of CIN and light conditions clearly contributed to improve the dynamics of the branching parameters. Focusing on nutrient absorption, the best values for the rate and branching density of roots with a cauline base of explants suggest that this root system might offer an efficient contact surface with the surrounding aquatic environment useful for innovative remediation technologies in open wastewater treatment systems [48,49].

Several aspects of adventitious root growth reviewed by [50] support the results generated in this work with rhizotrons in vitro: the experimental environment of in vitro rhizoclones with the root attached to the cauline base provided dynamic rhizogenic lines with antagonistic and synergistic speed and density indicators [9,15,16,17,51] that are promising for different purposes. Among the groups with isolated root architecture attached to a cauline base, T5L and T3L were distinguished as better in TRAM and DRAM. Additionally, the efficiency of TE and DLR from seedlings included T1L, T1O, T6L, and T6O. In prospect, each type of rhizogenic line of dynamically isolated adventitious roots of *Typha* represents a proliferation pathway for different technological processes of water rhizofiltration, with applications to the purification of water by the elimination of nutrients, metalloids, xenobiotics, and emerging pollutants. They can also replace or be complementary to water purification processes such as electrodialysis, distillation, ion exchange, reverse osmosis, and biodenitrification by reducing costs [52,53,54]. Furthermore, they can be useful in advanced oxidation processes applied to emerging micropollutants [55] and can be applied to innovative industrial bioprocesses, as aquatic herbaceous plants are candidates for producing high-value plant-based bioproducts used in biorefining technologies [56]. In basic research, isolated lines of *Typha* can feed back into the development of models of emerging species with the particular interest of delving into the dynamic plasticity of adventitious roots in the face of environmental stressors [9,51,57].

## 5. Conclusions

The adventitious roots of *T. domingensis* generated from seedlings and explants of isolated roots with cauline bases presented greater rooting dynamics under the influence of light. Particularly in the isolated roots attached to the cauline base, the dynamic processes that increased the growth rate and density of branching were activated with the contribution of CIN under light and dark conditions. It is uncertain whether the effect of BAP and other cytokinins at low concentrations may improve the dynamic rooting response. Among the six strains chosen, those from explants of roots with cauline bases offer great potential for scaling up to bioreactors owing to their lower architectural complexity. This technique is ideal for minimizing stress, saving energy, and maintaining water and nutrient intake during the purification of excess nutrients and other contaminants in wastewater. The next generation of technologies associated with wastewater treatment are challenged to reduce carbon emissions and reduce the use of physical and chemical nitrogen removal processes. Therefore, adventitious roots, as a complement to the secondary or tertiary processes of wastewater treatment, might contribute to addressing this challenge by intensifying the process and recovery of resources for higher-quality effluents. The next challenges are to establish cultures of adventitious roots and evaluate their removal efficiency of contaminants from contaminated water, which should allow for comparisons of their decontaminating power versus the cultivation of conventional plants.

## Figures and Tables

**Table 1:** Latin square design. Experiment 1 AIA-CIN concentrations vs. explant type.

Treatment	T1	T2	T3	T4	T5	T6	T7	T8	T9
CIN (mg/L)	0	0	0	0.1	0.1	0.1	1	1	1
AIA (mg/L)	0	0.1	1	0	0.1	1	0	0.1	1
Explant	Seed	Root	CBAS	Root	CBAS	Seed	CBAS	Seed	Root

**Table 2:** Average phenotypes related to the growth rate and contact surface of the root system of in vitro germinates of *T. domingensis* from Experiment 1 AIA-CIN.

			Contact Surface		Growth Rate		
	NR	LR	DRAM	DLR	TE	TRAM	TCR
AIA/CIN	Roots Plant^−1^	Root mm Plant^−1^	Root cm^−3^	Root mm cm^−3^	Root mm d^−1^	Root d^−1^	Root mm mm^−1^ d^−1^
0.0–0.0	11.67 ± 0.95 ^ª^	150.02 ± 9.94 ^a^	0.23 ± 0.02 ^a^	3.00 ± 0.20 ^ª^	5.57 ± 0.83 ^a^	0.62 ± 0.08 ^a^	0.26 ± 0.02 ^a^
0.0–0.1	5.00 ± 1.10 ^abc^	30.38 ± 2.85 ^ab^	0.10 ± 0.02 ^def^	0.61 ± 0.06 ^ab^	3.19 ± 0.35 ^c^	0.43 ± 0.06 ^bcd^	0.18 ± 0.02 ^c^
0.0–1.0	3.33 ± 0.56 ^bc^	27.30 ± 3.73 ^ab^	0.07 ± 0.01 ^ef^	0.55 ± 0.07 ^ab^	3.36 ± 0.26 ^c^	0.36 ± 0.05 ^bcd^	0.19 ± 0.01 ^bc^
0.0–10.0	3.00 ± 0.37 ^bc^	22.77 ± 2.10 ^b^	0.06 ± 0.01 ^ef^	0.46 ± 0.04 ^b^	3.23 ± 0.38 ^c^	0.40 ± 0.04 ^bcd^	0.18 ± 0.02 ^bc^
0.1–0.0	7.50 ± 1.28 ^abc^	90.97 ± 21.65 ^ab^	0.15 ± 0.03 ^bc^	1.82 ± 0.43 ^ab^	4.74 ± 0.59 ^ab^	0.52 ± 0.06 ^ab^	0.24 ± 0.02 ^ab^
0.1–0.1	7.33 ± 1.02 ^abc^	66.10 ± 15.61 ^ab^	0.14 ± 0.02 ^bc^	1.32 ± 0.31 ^ab^	3.67 ± 0.43 ^bc^	0.45± 0.08 ^abcd^	0.20 ± 0.02 ^abc^
0.1–1.0	7.67 ± 0.67 ^abc^	49.98 ± 3.47 ^ab^	0.15 ± 0.01 ^bc^	1.00 ± 0.07 ^ab^	3.29 ± 0.58 ^c^	0.38± 0.06 ^bcd^	0.18 ± 0.03 ^c^
0.1–10.0	3.17 ± 0.40 ^bc^	21.67 ± 2.33 ^b^	0.06 ± 0.01 ^ef^	0.43 ± 0.05 ^b^	2.86 ± 0.27 ^c^	0.40 ± 0.04 ^bcd^	0.17 ± 0.02 ^c^
1.0–0.0	8.50 ± 0.56 ^ab^	66.86 ± 8.07 ^ab^	0.17 ± 0.01 ^ab^	1.34 ± 0.16 ^ab^	3.36 ± 0.51 ^c^	0.52 ± 0.10 ^ab^	0.18 ± 0.03 ^bc^
1.0–0.1	6.80 ± 0.95 ^abc^	55.17 ± 16.13 ^ab^	0.14 ± 0.02 ^bcd^	1.10 ± 0.32 ^ab^	2.74 ± 0.23 ^c^	0.50 ± 0.10 ^abc^	0.16 ± 0.01 ^c^
1.0–1.0	5.33 ± 1.23 ^abc^	26.33 ± 4.21 ^ab^	0.11 ± 0.06 ^cde^	0.53 ± 0.08 ^ab^	2.83 ± 0.33 ^c^	0.33 ± 0.03 ^cd^	0.17 ± 0.02 ^c^
1.0–10.0	3.00 ± 0.45 ^bc^	26.45 ± 2.31 ^b^	0.06 ± 0.01 ^ef^	0.41 ± 0.05 ^b^	2.74 ± 0.41 ^c^	0.43 ± 0.06 ^bcd^	0.16 ± 0.02 ^c^
10.0–0.0	5.00 ± 0.86 ^abc^	54.62 ± 16.62 ^ab^	0.10 ± 0.02 ^def^	1.09 ± 0.33 ^ab^	3.47 ± 0.38 ^bc^	0.33 ± 0.03 ^cd^	0.20 ± 0.02 ^bc^
10.0–0.1	4.00 ± 0.93 ^abc^	32.81 ± 6.13 ^b^	0.08 ± 0.05 ^f^	0.66 ± 0.21 ^b^	2.90 ± 0.44 ^c^	0.31 ± 0.02 ^cd^	0.17 ± 0.02 ^c^
10.0–1.0	2.05 ± 0.22 ^bc^	23.78 ± 2.16 ^b^	0.05 ± 0.00 ^ef^	0.48 ± 0.04 ^b^	3.25 ± 0.31 ^c^	0.29 ± 0.00 ^d^	0.19 ± 0.02 ^bc^
10.0–10.0	2.33 ± 0.21 ^c^	20.33 ± 2.98 ^b^	0.05 ± 0.01 ^f^	0.40 ± 0.06 ^b^	2.84 ± 0.43 ^c^	0.31 ± 0.02 ^cd^	0.16 ± 0.02 ^c^

Different letters indicate groups of means with significant differences at the 0.05 level (Tukey’s multiple range test). NR = number of roots; LR = root length; DRAM = branching density; DLR = root length density; TE = elongation rate; TRAM = branching rate; TCR = relative growth rate; AIA = indoleacetic acid; CIN = kinetin.

**Table 3:** Average of phenotypes related to growth rate and contact surface of the root system of in vitro germinates of *T. domingensis* from Experiment 2 AIA-BAP.

			Contact Surface		Growth Rate		
	NR	LR	DRAM	DLR	TE	TRAM	TCR
AIA/BAP	Roots Plant^−1^	Root mm Plant^−1^	Root cm^−3^	Root mm cm^−3^	Root mm d^−1^	Root d^−1^	Root mm mm^−1^ d^−1^
0.0–0.0	11.33 ± 0.84 ^a^	84.18 ± 16.62 ^a^	0.23 ± 0.02 ^a^	1.68 ± 0.12 ^a^	5.43 ± 1.04	0.60 ± 0.10 ^a^	0.25 ± 0.03
0.0–0.1	3.04 ± 0.40 ^abc^	22.87 ± 0.83 ^ab^	0.06 ± 0.02 ^abc^	0.46 ± 0.02 ^ab^	3.27 ± 0.12	0.55 ± 0.06 ^abc^	0.19 ± 0.01
0.0–1.0	2.33 ± 0.21 ^bc^	19.25 ± 1.46 ^ab^	0.05 ± 0.01 ^bc^	0.39 ± 0.03 ^ab^	2.80 ± 0.20	0.33 ± 0.03 ^ef^	0.17 ± 0.01
0.0–10.0	2.50 ± 0.22 ^bc^	23.20 ± 2.03 ^ab^	0.05 ±0.01 ^bc^	0.46 ± 0.04 ^ab^	3.31 ± 0.29	0.36 ± 0.05 ^def^	0.02 ± 0.01
0.1–0.0	6.33 ± 0.67 ^ab^	40.78 ± 8.26 ^ab^	0.13 ± 0.03 ^ab^	0.82 ± 0.17 ^ab^	3.16 ± 0.36	0.48 ± 0.05 ^bcd^	0.18 ± 0.01
0.1–0.1	3.00 ± 0.37 ^abc^	20.20 ± 3.30 ^ab^	0.06 ± 0.02 ^abc^	0.40 ± 0.07 ^ab^	2.85 ± 0.46	0.40 ± 0.04 ^def^	0.16 ± 0.03
0.1–1.0	2.00 ± 0.00 ^c^	17.05 ± 1.96 ^b^	0.04 ± 0.00 ^c^	0.34 ±0.04 ^b^	2.44± 0.28	0.29 ± 0.00 ^f^	0.14 ± 0.02
0.1–10.0	2.67 ± 0.21 ^abc^	16.23 ± 1.42 ^b^	0.05 ± 0.01 ^abc^	0.32 ± 0.03 ^b^	2.32± 0.20	0.38 ± 0.03 ^ef^	0.14 ± 0.01
1.0–0.0	6.00 ± 0.58 ^ab^	83.92 ± 17.37 ^a^	0.12 ± 0.03 ^ab^	1.68 ± 0.35 ^a^	4.85 ± 1.08	0.57 ± 0.05 ^ab^	0.23 ± 0.02
1.0–0.1	2.17 ± 0.17 ^bc^	17.12 ± 1.94 ^b^	0.04 ± 0.01 ^bc^	0.34 ± 0.04 ^b^	2.52± 0.14	0.31 ± 0.02 ^ef^	0.15 ± 0.01
1.0–1.0	2.50 ± 0.22 ^abc^	19.78 ± 1.65 ^ab^	0.05 ± 0.01 ^abc^	0.40 ± 0.03 ^ab^	2.83 ± 0.24	0.43 ± 0.04 ^cde^	0.17 ± 0.01
1.0–10.0	2.23 ± 0.21 ^bc^	18.93 ± 0.99 ^ab^	0.05 ± 0.00 ^bc^	0.38 ± 0.02 ^ab^	2.67 ± 0.16	0.33 ± 0.05 ^ef^	0.16 ± 0.01
10.0–0.0	2.50 ± 0.34 ^abc^	17.40 ± 0.83 ^b^	0.05 ± 0.02 ^abc^	0.35 ± 0.02 ^b^	2.49 ± 0.12	0.38 ± 0.05 ^def^	0.15 ± 0.01
10.0–0.1	2.83 ± 0.31 ^abc^	21.18 ± 2.58 ^ab^	0.06 ± 0.00 ^abc^	0.42 ± 0.04 ^ab^	3.01 ± 0.36	0.38 ± 0.03 ^def^	0.17 ± 0.02
10.0–1.0	2.17 ± 0.17 ^bc^	17.93 ± 2.30 ^ab^	0.04 ± 0.01 ^bc^	0.36 ± 0.05 ^ab^	2.56 ± 0.33	0.31 ± 0.05 ^ef^	0.15 ± 0.02
10.0–10.0	2.50 ± 0.34 ^abc^	17.82 ± 3.11 ^b^	0.05 ± 0.01 ^abc^	0.36 ± 0.06 b	2.55 ± 0.44	0.33 ± 0.06 ^ef^	0.15 ± 0.02

Different letters indicate groups of means with significant differences at the 0.05 level (Tukey’s multiple range test). NR = number of roots; LR = root length; DRAM = branching density; DLR = root length density; TE = elongation rate; TRAM = branching rate; TCR = relative growth rate; AIA = indoleacetic acid; BAP = benzyl aminopurine.

**Table 4:** Effects of the AIA and CIN concentrations and the explant type on spatial–temporal dynamics of the phenotypic traits of adventitious roots in cultures under light and dark conditions.

Light (Exp 3)								
AIA	CIN	Explant	NR	LTR	TRAM	TE	DRAM	DLTR
0.0	0.0	Seed	9.60 ± 2.87	11.60 ± 3.58	0.34 ± 0.10	0.41 ± 0.13	0.19 ± 0.06	0.23 ± 0.07
0.0	0.1	Root	1.00 ± 0.00	0	0.04 ± 0.00	0	0.02 ± 0.00	0
0.0	1.0	CBAS	10.60 ± 1.69	9.76 ± 1.48	0.38 ± 0.06	0.35 ± 0.05	0.21 ± 0.03	0.20 ± 0.03
0.1	0.0	Root	1.00 ± 0.00	0	0.04 ± 0.00	0	0.02 ± 0.00	0
0.1	0.1	CBAS	11.40 ± 0.68	6.38 ± 0.79	0.41 ± 0.02	0.23 ± 0.03	0.23 ± 0.01	0.13 ± 0.02
0.1	1.0	Seed	11.40 ± 0.68	8.38 ± 0.71	0.41 ± 0.02	0.30 ± 0.03	0.23 ± 0.01	0.17 ± 0.01
1.0	0.0	CBAS	6.60 ± 1.57	2.34 ± 0.60	0.24 ± 0.06	0.08 ± 0.02	0.13 ± 0.03	0.05 ± 0.01
1.0	0.1	Seed	7.20 ± 1.11	3.18	0.26 ± 0.04	0.11 ± 0.03	0.14 ± 0.02	0.06 ± 0.01
1.0	1.0	Root	1.00 ± 0.00	0	0.04 ± 0.00	0	0.02 ± 0.00	0
Dark (Exp 4)								
0.0	0.0	Seed	11.00 ± 1.10	12.20 ± 1.64	0.39 ± 0.39	0.44 ± 0.06	0.22 ± 0.02	0.24 ± 0.03
0.0	0.1	Root	1.00 ± 0.00	0	0.04 ± 0.00	0	0.02 ± 0.00	0
0.0	1.0	CBAS	5.80 ± 2.44	5.30 ± 3.35	0.21 ± 0.09	0.19 ± 0.12	0.12 ± 0.05	0.11 ± 0.07
0.1	0.0	Root	1.00 ± 0.00	0	0.04 ± 0.00	0	0.02 ± 0.00	0
0.1	0.1	CBAS	9.20 ± 1.53	5.44 ± 0.90	0.33 ± 0.05	0.19 ± 0.03	0.18 ± 0.03	0.11 ± 0.02
0.1	1.0	Seed	11.4 ± 0.68	8.38 ± 0.71	0.41 ± 0.02	0.30 ± 0.03	0.23 ± 0.01	0.17 ± 0.01
1.0	0.0	CBAS	5.20 ± 2.27	4.38 ± 2.17	0.19 ± 0.08	0.16 ± 0.08	0.10 ± 0.05	0.09 ± 0.04
1.0	0.1	Seed	8.20 ± 2.35	5.32 ± 1.79	0.29 ± 0.08	0.19 ± 0.06	0.16 ± 0.05	0.11 ± 0.04
1.0	1.0	Root	1.00 ± 0.00	0	0.04 ± 0.00	0	0.02	0

Two Latin experimental designs were applied to study the principal effect of the AIA, CIN, and the explant type on rhizogenesis in rhizotron cultures under light (Exp 3) and darkness (Exp 4). Each rhizotron was inoculated with one explant type (seed, complete seedling; CBAS, SR with cauline base; root, isolated SR without cauline base) with five replicates by treatment (n = 45). TE = elongation rate; TRAM = branching rate; DRAM = branching density; DLTR = total root length density.

## Data Availability

Data is contained within the article.
